# The machinery of healthy vasoconstriction: an overview

**DOI:** 10.1007/s00424-025-03103-6

**Published:** 2025-07-11

**Authors:** Jana Pourová, Patrícia Dias, Milan Pour, Přemysl Mladěnka

**Affiliations:** 1 Department of Pharmacology and Toxicology, Faculty of Pharmacy, Charles University, Akademika Heyrovskeho 1203, 500 05 Hradec Kralove, Czech Republic; 2 Frick Center for Heart Failure and Arrhythmia, Dorothy M. Davis Heart and Lung Research Institute, College of Medicine, Ohio State University Wexner Medical Center, Columbus, OH USA; 3 Division of Pharmaceutics and Pharmacology, College of Pharmacy, Ohio State University, Columbus, OH USA; 4 Department of Organic and Bioorganic Chemistry, Faculty of Pharmacy, Charles University, Akademika Heyrovskeho 1203, 500 05 Hradec Kralove, Czech Republic

**Keywords:** Vasoconstriction, Calcium, PKC, RhoA/ROCK, EDCF

## Abstract

Tissue perfusion is acutely regulated by the changes in the vascular tone resulting in vasodilatation or vasoconstriction (there are also long-term changes in tissue perfusion, effectively accomplished by vascular remodeling). Even though vasodilatation predominates under physiological conditions, vasoconstriction represents an essential part of normal vascular physiology. The process of vasoconstriction is very complex, being influenced by many mediators, some of which are produced by the adjacent endothelial cells. The purpose of this review is to provide an overview of the machinery of vasoconstriction addressing the main components. First, the role of calcium is discussed including its intracellular and extracellular sources, its principal function in smooth muscle contraction machinery and mechanisms counteracting its effects. Subsequently, protein kinase C is included with its activation, effects and feedback. The role of RhoA/ROCK system is addressed in a similar way. The next section deals with the role of vascular endothelium-derived contracting factors and their effects on the adjacent smooth muscle cells. Finally, principal mechanisms of action of vasoconstrictive stimuli and myogenic tone are concisely discussed.

## Introduction

The primary role of the vascular system, crucial for the whole-body homeostasis, is to secure the transport of blood and substances to organs and tissues. The blood arteries are well adapted for this purpose. Briefly, the vessels are composed of three layers: *tunica intima*, known also as endothelium, formed by a single layer of endothelial cells in direct contact with the blood. Then, the adjacent layer, *tunica media,* mainly composed of vascular smooth muscle (VSM) cells and elastic fibers. And, finally, *tunica externa* (*adventitia*) that contains fibroblasts, nerve endings and adipocytes (perivascular adipose tissue, PVAT). In many vascular beds, glycocalyx formed by extracellular polysaccharides is present on the luminal surface [68]. The vascular system is capable of quick adaption to immediate needs of the organism. In general, the blood flow depends on the viscosity of the blood, on the length and diameter of the vessel, and on the pressure gradient. To regulate the flow, very rapid and highly effective change in the diameter of the blood vessels by vasoconstriction or vasodilatation is crucial. The more abundant small (feed) arteries and arterioles play a fundamental role in this process in contrast to the less abundant large (conduit) arteries. In the regulation of the arterial diameter, the VSM cells are the effectors which change the vessel diameter. The VSM cells tightly collaborate with endothelium, perivascular nerves, and PVAT. The endothelial cells are particularly important because they not only sense signals from the blood, but also produce certain vasoactive substances and participate in the modulation and propagation of the membrane potential. The process is significantly influenced by the presence of the intercellular gap junctions, which facilitate the synchronization of ion concentrations and the membrane potentials of adjacent cells as well as the transport of small signaling molecules. The capillaries are the vessels of the smallest diameter and differ from the others in many aspects. The capillary wall does not contain the VSM cells and is formed by one layer of endothelial cells, basal membrane and the pericytes. Among other functions, the pericytes participate in the regulation of microvascular tone [299]. All vascular cells can be influenced by a number of factors such as the membrane potential, vasoactive substances including neurotransmitters, pH changes, and physical stimuli. For this purpose, the cells dispose of diverse sensors such as plasma membrane ion channels and G-protein coupled receptors and intracellular receptors.

In addition to their different diameters, the blood vessels differ substantially in several aspects. First, vascular receptor distribution is not homogenous. Different vessel types display different receptor profiles and different levels of expression [161]. Distribution of these receptors depends on the vessel size, type (artery, capillary, vein) and location (CNS, coronary, periphery). The same is true for particular receptor subtypes (e.g. in mice, the vasodilatation to acetylcholine in coronary vessels is mediated by M_3_ and M_2_ receptors, while in the cerebral vessels by the M_5_ subtype [144, 301]). The resistance arteries, in contrast to the conduit arteries, display myogenic constriction (see Sect."Mechanical stimuli"). Second, the vessels fit the role of the corresponding tissue. While arteries in the skeletal muscles mainly secure adequate oxygen and nutrient supply during activity [75], the gut vessels form a part of the gut vascular barrier which facilitates the absorption of nutrients and protects against harm [29]. Accordingly, different mediators are produced in organ-specific way as was shown for endothelial cells [209]. By way of example, liver sinusoidal endothelial cells produce hepatocyte growth factor which promotes the hepatocyte survival and liver regeneration [151], while bone capillaries and VSM cells produce the bone morphogenic protein 2 that regulates bone formation [177]. Third, natural differences arise not only among species or specific vascular beds, but also between biological sexes [12, 123, 265]. The sex hormones and their receptors influence expression and activity of various ion channels including potassium [274, 311] and calcium [100, 240, 274, 277, 314] channels, maintenance of intracellular Ca^2+^ stores [74] (see Sect."SOCE"), and plasma membrane- [204, 272] and intracellular- [99, 160] ATPases. The differences in the regulation of the vascular tone represent one of the factors which determinate the differences in the incidence and course of cardiovascular diseases between sexes [265]. Notably, the menopause-related changes result also in changes in vascular signaling [154]. Fourth, other cell types come into play in a region-specific manner. In the CNS, the astrocytes play an important role in arterial tone regulation, and the same is true for microglia in the cerebral capillaries [53]. The brain pericytes participate in the neurovascular coupling that secures the local increase in the cerebral blood flow due to neuronal activity [87, 165]. The pericytes display several other functions, such as maintaining homeostasis in microcirculation, regulation of vascular growth and impact on immune function (for a review, see [2, 165, 299]). In the periphery, the myeloid cells have an impact on the vascular tone under hypoxia (reported in rats [196]) or inflammation (reported for inflammatory bowel disease [88]). In general, pathological conditions affect the vascular regulation, and reciprocally, the vascular dysregulation can lead to a dysfunction or even contribute to (or cause) a disease [13, 37, 273, 278].

The aim of this review is to summarize the machinery of vasoconstriction addressing its most important components in a comprehensive way. This topic is very broad and, in fact, other excellent reviews have been published [53, 90, 162, 225, 259, 276]. While the previous reviews often focused in a detail on a specific aspect, the primary goal of this paper is to demonstrate the complexity of the vascular contraction. The most important signaling pathways will be described, first on the level of the vascular smooth muscle, and then in orchestration with the endothelium. Finally, principal mechanisms of action of vasoactive stimuli will be discussed.

## Vascular smooth muscle in vasoconstriction

The blood vessels are permanently under the influence of both vasoconstrictive and vasodilatory stimuli; the prevailing factor determines the outcome. The VSM cell contraction is based on the interaction between two contractile proteins, actin and myosin, in the presence of sufficient Ca^2+^ amount in the cytoplasm. There are two principal sources of Ca^2+^ ions: release from the sarcoplasmic reticulum (SR) and influx of extracellular Ca^2+^ from Ca^2+^ channels. The Ca^2+^ ions bind to a cytoplasm protein called calmodulin and the resultant complex (Ca^2+^-CaM) activates myosin light chain kinase (MLCK). The MLCK phosphorylates myosin light chain (MLC) at the MLC_20_ subunit (Ser 19), allowing myosin to bind to actin, which initiates muscle contraction. Importantly, negative feedback mechanisms secure the transient nature of the responses evoked. At the intracellular level, a highly interconnected system of signaling pathways integrates various key structures.

Maintenance of the resting ion level ratios on both sides of the plasma membrane and resting membrane potential is secured by two important ATPases: Na^+^/K^+^-ATPase, and the plasma membrane Ca^2+^ ATPase (PMCA). The Na^+^/K^+^-ATPase is a protein, ubiquitously expressed in the plasma membranes of animal cells. The α_1_ isoform of the Na^+^/K^+^-ATPase is ubiquitous and plays a „housekeeping “ role, other isoforms are tissue specific with spatially restricted distribution. In the VSM cells, the α_2_ isoform was shown to be co-localized with the Na^+^/Ca^2+^ exchanger and with the K_ATP_ channels [76, 175, 176]. There are four subtypes of the PMCA, numbered from 1 to 4. The PMCA_1_ is a ubiquitous „housekeeping“ isoform, while the other isoforms are tissue specific, with PMCA_4_ having been reported in the VSM cells. The PMCA becomes activated upon binding of the Ca^2+^-CaM complex and has autoinhibitory properties [35]. Inside the VSM cells, transfer of Ca^2+^ ions between the stores in the SR and cytoplasm is most important. Transport from SR to cytoplasm is secured by the ryanodine receptor (RyR) and the inositol 1,4,5,-triphosphate receptor (IP_3_R) channels. There are three types of the RyR channels, named RyR_1_, RyR_2_ and RyR_3_ [145]. All three subtypes were found in the vascular smooth muscle [200], with a significant regional heterogeneity. The IP_3_R channels have a high degree of sequence and structural homology with the RyR channels but the former are more frequently expressed. Similar to the RyR channels, there are three isoforms, IP_3_R_1_, IP_3_R_2_ and IP_3_R_3_. The IP_3_R_1_ type is highly expressed in VSM and is abundant also in the endothelial cells [69, 264]. On the other hand, the sacro/endoplasmic reticulum calcium ATPase (SERCA) on the membrane of the SR uptakes cytosolic Ca^2+^ and decreases available cytosolic Ca^2+^. The SERCA in the VSM cells is activated by NO, which is produced in the adjacent endothelium and diffuses to the VSM cells [1].

For the sake of clarity, the following text will deal with principal vasoconstrictive signaling pathways according to its main components: calcium ions, protein kinase C, and the RhoA/ROCK system.

### Calcium ions

The Ca^2+^ ions are present in the extracellular space around the VSM cells, in their cytoplasm and in intracellular stores. There is a huge concentration gradient. In the resting state, the Ca^2+^ ion concentration outside the cell is ~ 2–3 mM, whereas it reaches approximately 100 nM [41, 98, 110] in the cytoplasm, ie. approx. twenty-thirty thousand times less. In the SR, calcium concentration may be subject to local deviations, because of uneven distribution of Ca^2+^-binding proteins. It is estimated to be 0.3–1 mM under the resting potential, several orders of magnitude higher than in the cytoplasm [41]. The movement of Ca^2+^ ions across membranes (i.e. from extracellular space or from the SR) results in a change in the plasma levels, which can be local or global, and sustained or transient (sparks) [5, 72, 110, 117, 233, 259]. Importantly, the local change means elevation of Ca^2+^ concentration just in the proximity of the Ca^2+^-sensitive cellular structures such as the big conductance calcium-activated K^+^ channels (BK_Ca_). Colocalization of the participating components is important [259]. Due to K^+^ efflux, the membrane potential becomes more negative, which can result in vasodilatation. By contrast, a global increase in the cytosolic calcium concentration ([Ca^2+^]_c_) triggers vasoconstriction (see Sect."Effects of Ca^2+^"). Last but not least, the changes in Ca^2+^ levels can also take the form of waves (see Sect."The Ca^2+^ feedback").

#### Intracellular Ca^2+^ sources

The SR is the main intracellular store of Ca^2+^ ions. This organelle is present in all types of the VSM cells and constitutes a larger proportion of the total cell volume in the large elastic arteries compared to their small counterparts [26, 162]. The capacity of the SR is enhanced by the presence of the Ca^2+^-binding proteins, such as calsequestrin, calreticulin or calnexin. Ca^2+^ ions are released from the SR via the the RyR and IP_3_R channels. The Ca^2+^-induced Ca^2+^ release (CICR) is triggered at the concentration of Ca^2+^  ~ 3 µM [162] in the proximity of the SR. This amplification is an important component of calcium signaling; however, it may not be essential for vasoconstriction [89]. On the other hand, the CICR is extremely important for activating the BK_Ca_ channels and for the negative feedback after excessive depolarization and calcium increase (see Sect."The Ca^2+^ feedback") [5]. The CICR is promoted by the activators of RyR and IP_3_R channels, and, surprisingly, also by cAMP [234]. The Mg^2+^ ions or procaine exert the opposite effect as well as an excessive amount of calcium (high micromolar concentrations) [276].

The Ca^2+^ can also enter other organelles, mainly the mitochondria and the lysosomes through specific structures, such as mitochondria-associated membranes (MAMs) [222]. Calcium up-take by other cell organelles comes into play if the Ca^2+^ concentration in their proximity is abnormally high (for mitochondria >  ~ 5–10 μM). The stored Ca^2+^ can be effluxed back to the cytoplasm and modulate [Ca^2+^]_c_. The mitochondrial Ca^2+^ buffering may become more important under pathological conditions, such as atherosclerosis [66].

#### Extracellular Ca^2+^ sources

Extracellular calcium ions enter the cells through either the voltage-gated (Ca_v_) channels or most of the types of the transient receptor potential (TRP) channels. In the VSM cells, two types of the voltage-gated Ca_v_ channels (Ca_v_3.x or T-type and Ca_v_1.2 or L-type) and various TRP channel subtypes have been identified (TRPC_1/3/4/5/6_, TRPV_1/2/3/4_, TRPA_1_, TRPM_4/8_ and TRPP_1_) (for a review, see [55] and [61]). The entry of extracellular Ca^2+^ occurs continuously as a part of the basal ion transfer. As the Ca^2+^ ions are concomitantly being removed from the cytoplasm into the intracellular stores (SERCA), and the Ca^2+^ efflux through the plasma membrane (PMCA, Na^+^/Ca^2+^ exchanger NCX) is operating, this basal ion transfer leaves the VSM cells in the resting state. The Ca_v_ channels open upon depolarization of the plasma membrane and are regarded as the principal source of Ca^2+^ for vasoconstriction. The TRP channels are activated by various stimuli according to the channel subtype, such as the binding of agonists to the G protein-coupled receptor (GPCR) of G_q_-type, increased pressure and wall stress (greater than ~ 20%) causing myogenic depolarization (see Sect."Mechanical stimuli"), and thermal stimuli (see Sect."Thermal stimuli").

#### SOCE

The intracellular store Ca^2+^ depletion can result in extracellular Ca^2+^ influx called store-operated Ca^2+^ entry (SOCE) [214]. Substantial Ca^2+^ depletion is required for SOCE initiation, and constitutive Ca^2+^ leakage has no effect. However, inhibition of the Ca^2+^ re-uptake by SERCA is a sufficient stimulus in itself [214]. On the other hand, SOCE becomes fully activated before the SR is completely depleted which can be considered a safety mechanism against a complete loss of stored Ca^2+^. The SOCE is regulated by various proteins such as calmodulin and calcium-calmodulin-dependent protein kinase II (CaMKII) [20, 283]. While the primary purpose of SOCE is the refilling of the intracellular Ca^2+^ stores, it is also involved in other vascular processes such as permeability regulation, vascular repair, cell proliferation, and immune response [190]. The SOCE is operating not only in the VSM cells but also in the adjacent endothelium (see Sect."Endothelium in vasoconstriction"). The primary purpose of endothelial SOCE remains the same (e.i. refilling of the intracellular Ca^2+^ stores), but additional specific functions have been attributed including the role in the endothelial nitric oxide signaling and blood pressure control [48, 191], endothelial permeability [121, 256], endothelial oxidative stress response [38, 139, 185], and excitation-transcription coupling [24, 63, 226].

Two principal proteins are involved in SOCE—the stromal interaction molecule (STIM) within the SR cisternae, and Orai protein located on the plasma membrane. The STIM acts as a Ca^2+^ sensor in the SR [231]. Two homologues, STIM_1_ and STIM_2_, are known, and the STIM_1_ seems to be the principal one in SOCE. Similarly, three types of the Orai protein have been described, with Orai_1_ being the most important. First, due to Ca^2+^ depletion below certain threshold, the STIM oligomerizes in the SR membrane. Subsequently, the STIM translocates and interacts with the plasma membrane lipids (phosphatidylinositol 4,5-bisphosphate (PIP_2_)), to activate the Orai proteins through protein–protein interaction. The four Orai_1_ subunits form a Ca^2+^-release-activated Ca^2+^ channel (CRAC) for extracellular Ca^2+^ entry and several channels assemble into a cluster that forms the SR-plasma membrane nanojunctions [52, 59]. The Ca^2+^ current (I_CRAC_) is a highly Ca^2+^-selective low-conductance current which can be terminated by intracellular Ca^2+^ ions. Surprisingly, IP_3_R channels (i.e. important channels for Ca^2+^ depletion from the SR) modulate the STIM/Orai interaction [58]. In addition to the I_CRAC_, the intracellular store Ca^2+^ depletion can result in store-operated (I_SOC_) and I_CRAC_-like currents with different electrophysiological properties. Regarding the I_SOC_ current, the Orai_1_ mediated Ca^2+^ entry causes TRPC_1_ channel incorporation into the plasma membrane in close apposition to Orai_1_ enabling the TRPC_1_ activation by the STIM_1_ and providing another Ca^2+^ (and, possibly, also Na^+^) point of entry [166, 231, 296]. The TRPC_1_ channels are the principal channels for the vascular I_SOC_, but other channel subtypes (e.g. TRPC_4_ and TRPC_5_) also participate, at least in some vascular beds [6, 19, 93, 212, 300]. As regards the I_CRAC_-like current, the Orai_1_ and TRPC_1_ channels (and possibly other TRPC channels) can assemble into a heteromeric channel with mixed properties between I_CRAC_ and I_SOC_ [190]. While the interactions among STIM_1_, Orai_1_ and TRPC_1_ are considered principal in SOCE, additional options also exist, such as the STIM_1_-mediated TRPC_1_-based I_SOC_ in the native contractile VSMCs which was Orai_1_-independent and involved interactions between protein kinase C (see Sect."Protein kinase C") and PIP_2_ [172]. In endothelium, the NO production may be selectively coupled to Orai_1_ via TRPC_4_ [39] but not TRPC_1_ channels [190, 249]. Different ways of intracellular Ca^2+^ store refilling can dominate according to species and vascular beds. Noteworthy, SOCE research can be strongly influenced by the use of freshly isolated or long-term cultured VSM cells [172]. In the former case, the native contractile cells display Ca^2+^ currents which correspond to the TRPC-based SOCE and low expression of the Orai_1_ proteins. In contrast, the long-term cultured cells display a non-contractile phenotype with proliferative, migrative and growth characteristics and the Ca^2+^ entry corresponding rather to the I_CRAC_ [17, 21, 213]. In other words, it is crucial to define the cell phenotype when studying the vascular SOCE.

The dysregulation of SOCE plays an important role in the development of vascular alterations or even diseases including hypertension [23], pulmonary hypertension [36, 235] and atherosclerosis [47, 313]. Accordingly, the expression of STIM_1_ and Orai_1_ in human microcirculation (reported in mesenteric artery) increased with age and negatively correlated with impaired endothelium-dependent vasodilatation [57]. The CRAC channel activation was increased in aortas from stroke-prone spontaneously hypertensive rats compared to normotensive animals by mechanisms dependent on STIM_1_ and Orai_1_ activation [73]. The mutation in STIM_1_ and Orai_1_ is likely related to many diseases (for review, see [141]). The same may be true for modulators of SOCE such as IP_3_R channels [108].

#### Effects of Ca^2+^

Membrane depolarization and global increase of the cytosol Ca^2+^ concentration initiate the vascular smooth muscle cell constriction. The constriction cascade is triggered if [Ca^2+^]_c_ in the VSM cells exceeds certain value (in the resting state, the global calcium concentration is approx. 100 nM, depolarization of the plasma membrane by ~ 15 mV from the resting potential elevates global Ca^2+^ levels to ~ 300–400 nM [98]). The Ca^2+^ ions act either in complex with the regulatory proteins (principally with calmodulin), or directly. The Ca^2+^-CaM complex changes the conformation of MLCK and thus activates this kinase [41, 101] with subsequent phosphorylation of MLC. Vasoconstriction is the result of the myosin-actin interaction. The Ca^2+^-CaM complex also activates phosphatidylinositol-4,5-bisphosphate 3-kinase (PI_3_K) [247], which sets in motion the vasoconstrictive RhoA/ROCK system (see Sect."RhoA/ROCK system"). These principal events are supported by other pro-vasoconstrictive effects, such as the Ca^2+^-CaM complex binding to calponin [162] which prevents its binding to actin (actin can thus bind to myosin).

In parallel, the elevated [Ca^2+^]_c_ influences the ion transfer across membranes (both plasmatic and intracellular), which, in turn, can modify the membrane potential. The transfer of Ca^2+^ and K^+^ ions is the most important. The transfer of Ca^2+^ into the cytosol intensifies the [Ca^2+^]_c_ elevation while the K^+^ ions are principal for the membrane potential modification. On the SR, the Ca^2+^ ions activate the IP_3_R and the RyR channels. To open the IP_3_R channels, both Ca^2+^ and inositol trisphosphate (IP_3_) are required (IP_3_ concentration of 10 μM induced a rapid and huge Ca^2+^ release in the primary cultured rat aortic smooth muscle cells [304]). Subsequently, amplification of the Ca^2+^ signals through the Ca^2+^-induced Ca^2+^ release (CICR) is allowed [42, 91, 239, 264, 276]. The IP_3_R [275] and the RyR channels are also opened by CaMKII which has been previously activated by the Ca^2+^-CaM complex [65, 91, 270]. Another complex, Ca^2+^-S100A1, activates the RyR and Ca_v_1.2 channels. The Ca^2+^-S100A1 and the Ca^2+^-CaM complexes bind to the same site on the RyR receptors [298] but have opposite effects at higher Ca^2+^ concentrations (> 1 µM) [182]. Importantly, the Ca^2+^ ions display the effects only within certain concentrations. The RyR channels are activated at the Ca^2+^ concentration ranging roughly from hundreds of nM to units of μM with the amplification of the Ca^2+^ signal (CICR) [61, 91, 150, 276]. Similarly, the activity of the IP_3_R channels is inhibited by an abnormally high Ca^2+^ level in their proximity [91, 275]. The channel inactivation likely results from presence of several distinct Ca^2+^ binding sites. The bell-shaped response to Ca^2+^ prevents excessive contraction, and is an important part of vascular physiology. Importantly, the IP_3_R and the RyR channels cannot be understood as strictly pro-vasoconstrictive channels. The same channels participate in calcium sparks and CICR with only local elevations in the Ca^2+^ concentrations which result in a vasodilatory action (see Sect."The Ca^2+^ feedback").

On the plasma membrane, the Ca^2+^ ions temporarily inactivate the K_v_ channels [44], which contributes to its depolarization and maintaining the Ca_v_ channels in an open-state (latter, when depolarization reaches a certain value, the K_v_ channels are opened and return the plasma membrane to the resting membrane potential). In some vascular beds, the opening of the Cl_Ca_ channels with Cl^−^ efflux contributes to depolarization [147]. The Ca^2+^-CaM complex activates calcineurin (protein phosphatase 2B), which inhibits the K_ATP_ channels on the plasma membrane and hence also contributes to its depolarization [228]. Finally, the effects of Ca^2+^ on the vascular TRP channels are noteworthy [103, 149]. In vascular systems, TRPC_3_, TRPC_6_, TRPM_4_, and TRPM_5_ are activated by Ca^2+^ionts [28]. Depending on the channel subtype, the effects of the Ca^2+^ ions can result in channel stimulation or inhibition. In general, these effects are mediated directly by Ca^2+^ or (more frequently) involve the Ca^2+^-binding proteins with calmodulin being the most prominent one. The calmodulin-binding domains have been identified in the cytosolic regions of various TRP channels, and these sites can bind both the free-calmodulin and the Ca^2+^-CaM complex. The third mechanism, by which the Ca^2+^ ions influence the TRP channels is through the activation of phospholipase C with the hydrolysis of PIP_2_, and the production of inositol 1,4,5-triphosphate and diacylglycerol. For most TRP channels, PIP_2_ acts as a positive regulator, and the PIP_2_ hydrolysis leads to channel desensitization [279]. The role of vascular TRP channels may differ among vascular beds and species.

#### The Ca^2+^ feedback

The mechanisms counteracting vasoconstriction are physiologically activated from certain [Ca^2+^]_c_ levels (approximately at higher micromolar range). Determination of the exact critical concentration is not trivial, however, its individual value in the proximity of a particular cellular structure is crucial. The underlying mechanism is based on the modulation of the ion channel status, on the recovery of [Ca^2+^]_c_ and the membrane potential to the resting state. The Ca^2+^ ions act either directly, or in complex with regulatory proteins. On the plasma membrane, the Ca^2+^ ions activate the BK_Ca_ channels with K^+^ efflux and the membrane potential becomes more negative (hyperpolarization) [118]. As a consequence, the voltage-gated Ca_v_ channels on the plasma membrane are indirectly inactivated, and the influx of extracellular Ca^2+^ prevented (in the vascular smooth muscle, the Ca_v_3.2 channel subtype is prominent [95]). On the SR, the IP_3_R and the RyR channels are also inactivated from certain [Ca^2+^]_c_. Inhibition of these channels stops CICR [65, 91]. Inhibition of the RyR channels is mediated by the Ca^2+^-CaM complex. A similar effect is exhibited by another calcium-binding protein, sorcin [52]. The decrease of [Ca^2+^]_c_ to the resting values is secured by several mechanisms. The Ca^2+^-CaM complex activates plasma membrane calcium ATPase (PMCA) [35]. The Ca^2+^ ions activate the CaMKII which phosphorylates the regulatory protein phospholamban (Thr-17) and, in this way, the SERCA with Ca^2+^ re-uptake to the SR is activated [43]. Notably, a certain part of the cytosolic Ca^2+^ can be transferred to mitochondria (see Sect."Intracellular Ca^2+^ sources").

The negative feedback regulation of the vascular tone includes calcium sparks. These local sparks originate from the SR through the clusters of the RyR channels, and the signal is amplified by CICR. The calcium sparks cause a local elevation in the Ca^2+^ concentration (10–100 μM), and, paradoxically, result in vasodilatation in the resistance arteries. The sparks are self-limiting as maximal activity of the RyR channels is at Ca^2+^ concentration ~ 10 μM, and its further increase decreases the probability of their opening. On the other hand, the RyR channels are closed at Ca^2+^ concentration below ~ 100 nM [61, 276]. Local increase in Ca^2+^ leads to the activation of Ca^2+^ gated channels, including the BK_Ca_ channels, resulting in the K^+^ efflux. This efflux leads to plasma membrane hyperpolarization (more negative) and inactivation of the voltage-gated channels, including the Ca_v_ channels (the Ca_v_1.2. channels are the principal source of extracellular Ca^2+^). The global [Ca^2+^]_c_ remains at resting level or decreases, giving rise to vasodilatation. Importantly, to function properly, the calcium sparks occur locally very close to the plasma membrane, and physical proximity of the SR and the BK_Ca_ and Ca_v_ channels is necessary [259]. Also importantly, consecutive calcium sparks can form a calcium wave, which is more likely to occur in the arterioles then in the arteries. If such a wave results in a global [Ca^2+^]_c_ elevation, vasoconstriction follows [5]. The calcium waves (intracellular or intercellular) can have oscillatory character (i.e. during vasomotion [189]).

The calcium feedback mechanisms vary across different vascular beds. There are differences between small arterioles and upstream bigger arteries [289], as well as regional differences among arterioles. As regard the latter, while the Ca^2+^ sparks generally support vasodilatation, the myogenic tone was promoted in isolated retinal arterioles (25–40 μm external diameter) [140]. In some vessels, the calcium sparks can open the Cl_Ca_ channels formed by the TMEM16A protein. In the VSM, the opening of these channels leads to Cl^−^ efflux from the cell and membrane depolarization [106, 147]. Up-regulation of the Cl_Ca_ channels was described under hypertension, and accounts, at least partially, for the endothelial dysfunction [170]. In general, the intracellular calcium handling is altered with aging and can be related to cardiovascular diseases [92].

**Fig. 1:**
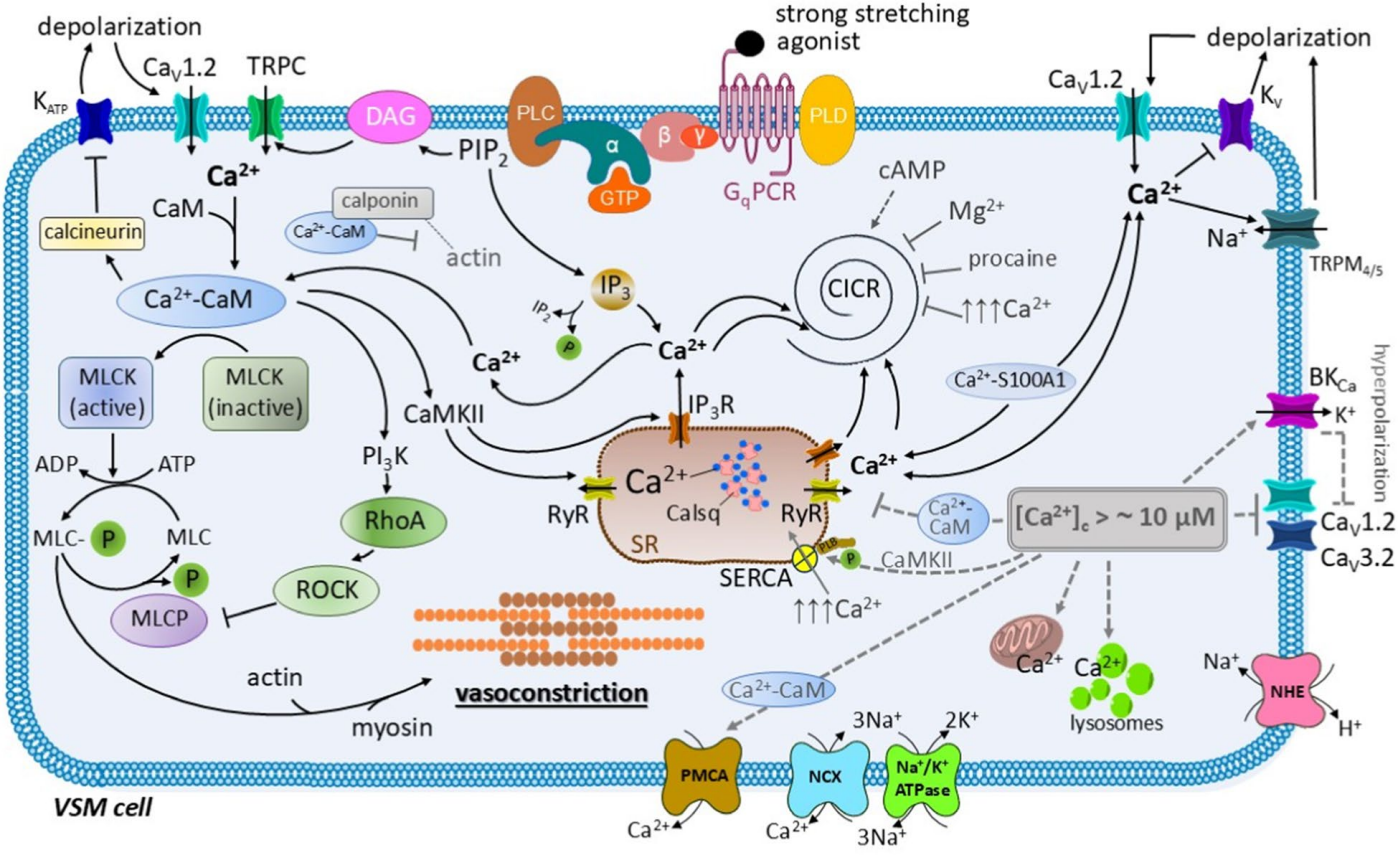
Simplified role of calcium in vascular smooth muscle (VSM) cells. Ca^2+^ ions are stored in the sarcoplasmic reticulum (SR) bound to the protein calsequestrin (Calsq) and, at lower levels, in mitochondria and lysosomes. The Ca^2+^ ions are released from the SR via inositol trisphosphate (IP_3_Rs) and ryanodine (RyRs) channels. Increase in Ca^2+^ concentration near to the SR gives rise to further calcium-induced calcium release (CICR). The CICR, promoted by cAMP, mediates signal amplification. A rise in the cytoplasmatic Ca^2+^ concentration can also be triggered by several stimuli, including the strong stretching of the vessel wall or the binding of an agonist to the G protein-coupled receptor of the G_q_ type (G_q_PCR). The G_q_PCR agonist leads to the activation of phospholipase C (PLC) and cleavage of phosphatidylinositol bisphosphate (PIP_2_) into inositol trisphosphate (IP_3_) and diacylglycerol (DAG), which, in turn, promote the release of Ca^2+^ from the SR and the activation of the transient receptor potential canonical (TRPC) channels, respectively. The Ca^2+^ influx also occurs through the Ca_v_1.2 (L-type calcium channels). Once in the cytoplasm, the Ca^2+^ ions exhibit their effects either directly or bound to proteins, mainly as the calcium-calmodulin (Ca^2+^-CaM) and Ca^2+^-S100A1 complexes. The direct effects of the Ca^2+^ ions include: 1) activation of RyRs on the SR with subsequent induction of the CICR; 2) activation of theTRPM_4_ and TRPM_5_ channels on the plasma membrane with Na^+^ influx, depolarization and increase in the activity of the Ca_v_1.2 channels; 3) inactivation of the voltage-gated K^+^ channels (K_v_) on the plasma membrane maintaining the VSM cell depolarization. The Ca^2+^-CaM complex: 1) activates the myosin light chain kinase (MLCK) with subsequent phosphorylation of the myosin light chain (MLC) and initiation of contraction; 2) activates phosphatidylinositol-3-kinase (PI_3_K) which subsequently activates the RhoA/ROCK system; 3) binds to calponin and prevents its binding to actin; 4) activates calcineurin, which inhibits the ATP-dependent K^+^ (K_ATP_) channels; 5) activates the calcium-calmodulin-dependent protein kinase II (CaMKII) which in turn activates IP_3_Rs and RyRs on the SR. The Ca^2+^-S100A1 complex activates RyRs on the SR and Ca_v_1.2 channels. A negative feedback loop is triggered at calcium concentrations of low tens of µM. At these concentrations, the Ca^2+^ ions: 1) activate the large conductance calcium-activated potassium channels (BK_Ca_) with the consequent inactivation of the Ca_v_1.2 (L-type) and Ca_v_3.2 (T-type) calcium channels on the plasma membrane and 2) activate the CaMKII. The kinase activates the sarco/endoplasmic reticulum calcium-ATPase (SERCA) through the phosphorylation of the regulatory protein phospholamban (PLB), with the consequent transport of calcium from the cytosol into the SR. At Ca^2+^ concentrations higher than 10 µM, the Ca^2+^-CaM complex 1) inactivates the RyRs on the SR and terminates the CICR, and 2) activates the plasma membrane calcium ATPase (PMCA). Other mechanisms securing calcium removal include the sodium/calcium exchange (NCX) and the transfer of Ca^2+^ ions into the mitochondria. The Ca^2+^ sparks operate in the same modus. Local Ca^2+^ increase activates the BK_Ca_ channels, the efflux of K^+^ leads to hyperpolarization and Ca_v_ channel inactivation. Other abbreviations: ADP, adenosine diphosphate; ATP, adenosine triphosphate; [Ca^2+^]_c_, cytosolic calcium concentration; CaM, calmodulin; cAMP, cyclic adenosine monophosphate; GTP, guanosine triphosphate; IP_2_, inositol 1,4-bisphosphate; MLC-P, myosin light chain phosphorylated; MLCP, myosin light chain phosphatase; Na^+^/K^+^-ATPase, sodium/potassium ATPase; NHE, sodium/proton exchanger; P, phosphate; PLD, phospholipase D; ROCK, Rho-associated protein kinase

The role of Ca^2+^ is summarized in Fig. 1.

### Protein kinase C

Protein kinase C (PKC) is a widely distributed cytoplasmic serine/threonine kinase with many isoforms [7]. The isoforms differ in their dependence on Ca^2+^ during activation. While conventional cPKCs (α, β, γ) require Ca^2+^, diacylglycerol and phosphatidylserine to be activated, just diacylglycerol and phosphatidylserine are needed to activate the novel nPKCs (δ, ε, η, θ), and, finally, the atypical aPKCs are activated by phosphatidylserine itself. Expression of individual isoforms in the VSM cells differs among species [85], and the precise role of each isoform is not yet fully understood. In the human VSM, the PKC_α_,PKC_β_,PKC_δ_,PKC_ε_ isoforms have been identified [85].

#### Activation of PKC

The principal way of the PKC activation in the VSM cells is the activation of the GPCR of the G_q_-type, and the production of diacylglycerol (DAG) and IP_3_. First, the GPCR of the G_q_-type is activated by an agonist or due to mechanical stimulation [7, 180, 252]. This activation results in the dissociation of the α subunit of the G-protein followed by the activation of phospholipase C (PLC) which subsequently hydrolyses the membrane phospholipid PIP_2_ to DAG and IP_3_. Phospholipase D is activated in a similar way and hydrolyses phosphatidylcholine to DAG and choline. DAG and IP_3_ are important second messengers involved in the PKC cascade. The lipophilic DAG is localized in the proximity of the plasma membrane, where the substance activates the PKC with the participation of phosphatidylserine and Ca^2+^ ions (conventional PKCs) [86]. This activation is a complex process and involves PKC phosphorylation by the PKC kinase and autophosphorylation followed by the translocation to the plasma membrane with the participation of annexins [51] and other proteins [230]. The molecules of IP_3_ are water soluble and diffuse through the cytosol to the SR surface, where the IP_3_R channels are activated with concomitant Ca^2+^ release [254]. Amplification of the Ca^2+^ signal (CICR) follows (see Sect."Effects of Ca^2+^"). Both DAG and IP_3_ have additional effects, related to vasoconstriction. On the plasma membrane, the TRPC_6_ channels are directly activated by DAG [237], and the TRPC_3_ channels by IP_3_ [255, 318]. Both channels mediate extracellular ion influx (primarily Ca^2+^, but also Na^+^) resulting in membrane depolarization with opening of the voltage-gated channels and [Ca^2+^]_c_ increase. The DAG can be converted by DAG lipase into arachidonic acid, which inactivates the myosin light chain phosphatase (MLCP) both directly (at micromolar concentrations), and via the activation of ROCK (see Sect."Activation of RhoA and ROCK") [10, 82]. IP_3_ is rapidly cleaved by specific phosphatases to inositol 1,4-bisphosphate (IP_2_) [285].

Other ways of PKC activation exist. The PIP_2_ can be phosphorylated by PI_3_K to yield another second messenger, phosphatidylinositol-3,4,5-triphosphate (PIP_3_) which activates the PKC [251]. In addition, PI_3_K contributes to the activation of the pro-vasoconstrictive RhoA/ROCK pathway (see Sect."Activation of RhoA and ROCK"). The PI_3_K has already been mentioned—it is activated by the Ca^2+^-CaM (see Sect."Effects of Ca^2+^"). PKC can also be cleaved by the caspases, generating a catalytically active kinase domain, or activated by lipid cofactors such as ceramide or arachidonic acid, or through lipid-independent mechanisms, such as oxidative modifications or tyrosine nitration [257]. Direct activation of PKC can be induced by phorbol esters [130]. Noteworthy, the PKC activation is modulated by vasodilatory mediators produced by the adjacent endothelium as demonstrated for H_2_O_2_ [84], and in the case of PKCα [111] and PKC_ε_ [16] also for NO.

#### Effects of activated PKC

The active PKC displays multiple effects in the VSM cells through the phosphorylation of target structures [225]. In general, the kinase causes depolarization of the plasma membrane, rise of [Ca^2+^]_c_, inactivation of MLCP and vasoconstriction. On the plasma membrane, effects on ion transfers are crucial. PKC activates the Ca_v_1.2 channels both directly and indirectly via activated tyrosine kinase SRC (interestingly, the Ca_v_1.2 channels can also be activated directly by PI_3_K, which activates PKC (see Sect."Activation of PKC") [31, 251]), and inhibits the K^+^ channels, principally the BK_Ca_ [153, 186, 261] and K_v_ channels [30, 201], but also the K_ATP_ channels [224, 228, 243]. Via this mechanism, PKC promotes depolarization and secondarily increases the activity of the voltage-gated channels including Ca_v_1.2. PKC also inhibits Na^+^/K^+^ ATPase [22] and activates the Na^+^/H^+^ exchanger [14, 15] with subsequent depolarization and alkalinization of the cytoplasm [297].

Inside the VSM cells, the PKC inhibits MLCP and hence prevents the dephosphorylation of myosin [236, 295]. The MLCP contains a phosphatase (PP1c) and myosin phosphatase target (MYPT1) subunits. Dissociation of the PP1c–MYPT1 complex causes MLCP inactivation. The activity of MLCP is regulated by two endogenous inhibitors, protein kinase C-dependent phosphatase inhibitor of 17 kDa (CPI-17) and phosphatase holoenzyme inhibitor (PHI-1, member of the CPI-17 family). In the MLCP inactivation, PKC participates in two ways: phosphorylation of MYPT1 and PKC/arachidonic acid-induced dissociation of the PP1c-MYPT1 complex [4, 82] or the activation of CPI-17, PHI-1 and arachidonic acid and dissociation of the PP1c–MYPT1 complex [4]. In addition to the MLCP inhibition, PKC inhibits soluble guanylate cyclase and thus cGMP formation, and NO-induced vasodilatation [122]. Last, PKC phosphorylates proteins that bind actin in their unphosphorylated forms, such as calponin and caldesmon [131]. Other kinases may also be involved in calponin phosphorylation [302]. Noteworthy, autophosphorylation of PKC is calponin-dependent [133]. Accordingly, calponin is able to activate PKC in vitro; the knock-down of the calponin gene inhibited the PKC-dependent contraction [120, 131, 132, 225].

The role of PKC in arterial contraction is more prominent in small resistance arteries compared to larger arteries, where other pathways like Rho/ROCK also play a significant role [135]. Alterations in the PKC activity are implied in the pathogenesis of a number of diseases including systemic and pulmonary hypertension, diabetic vasculopathy, atherosclerosis, vasospasm and many others (for reviews, see [184, 225]). The PKC inhibitors are widely used in basic research, and have also various clinical applications [156] including the vessel-related pathologies (in some cases with inconclusive results [50]).

#### The PKC feedback

Activation of PKC is usually regarded as supportive of vasoconstriction. However, the kinase regulates its own effects and ensures transient contraction. The active PKC inactivates the MLCK and phosphorylates MLC_20_, which limits interaction between myosin and actin [7, 112]; activates PMCA on the plasma membrane and SERCA on the SR with decrease of [Ca^2+^]_c_ [162, 236]; and facilitates the dissociation of G_αi_-GTP from adenylate cyclase, thereby terminating its inhibition [126]. Notably, the PLC is inactivated by protein kinases A and G which both mediate vasodilatation [197].

Additionally, the PKC may increase the synthesis of the GTPase-accelerating protein (GAP) [171], which is involved in the inactivation of the pro-vasoconstrictive RhoA-GTP (see Sect."Activation of RhoA and ROCK"). In the endothelial cells (see Sect."Endothelium in vasoconstriction"), the active PKC stimulates the production of vasodilatory NO [206] and the secretion of natriuretic peptide C [183]. The PKC might also activate the Na^+^/Ca^2+^ exchanger (NCX) [253], and inhibit the TRPC channels involved in SOCE [242], but evidence in the VSM is lacking.

**Fig. 2:**
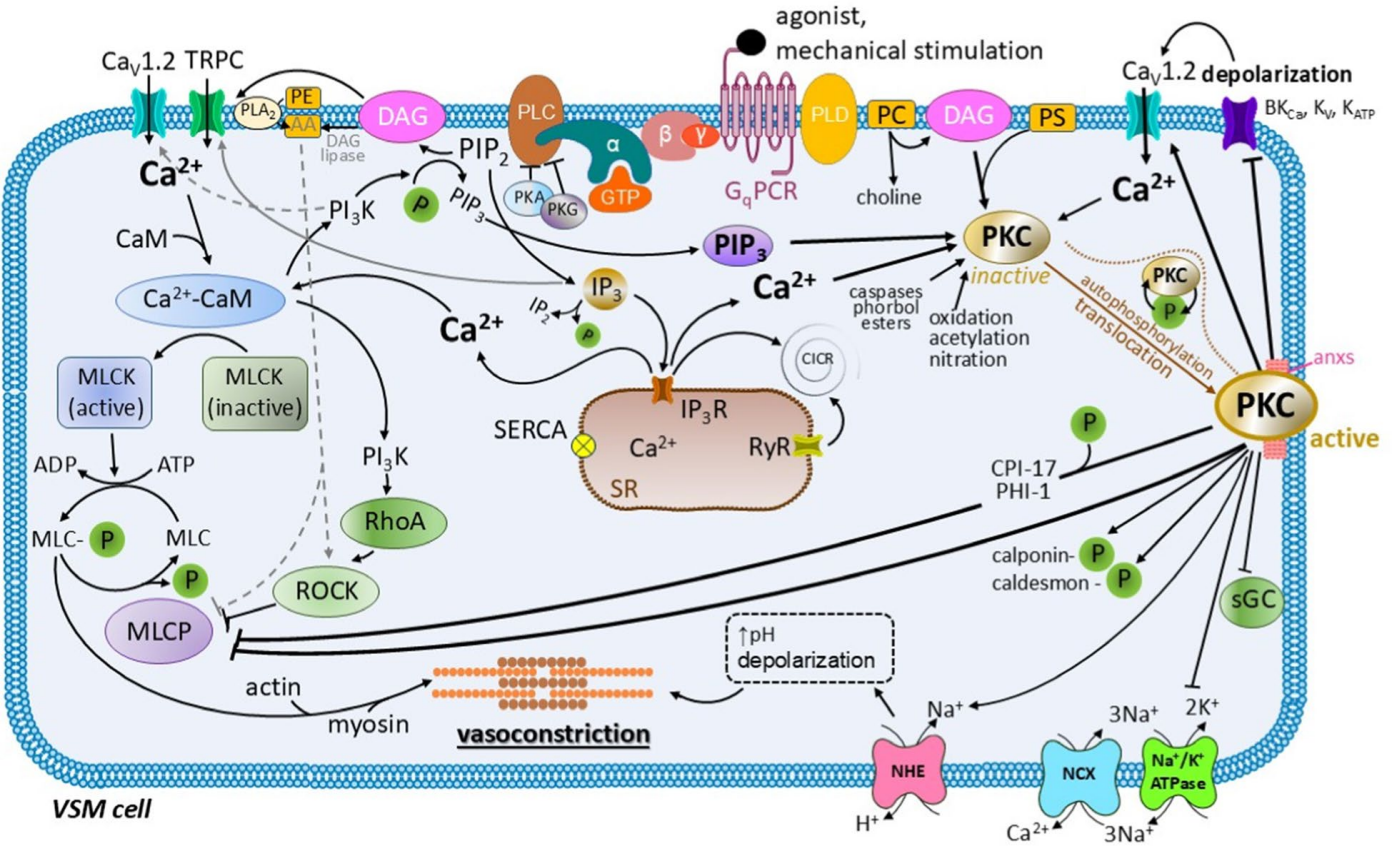
The role of protein kinase C (PKC) in the vascular smooth muscle (VSM) cell contraction. The activation of PKC occurs through the action of vasoconstrictors on the G protein-coupled receptor of the G_q_ type (G_q_PCR) on the plasma membrane, or by mechanical stimulation of the “stretch-sensitive” receptors. Upon binding of an agonist to the G_q_PCR, the G-protein α subunit dissociates and the activation of phospholipase C (PLC) takes place. Afterwards, PLC hydrolyses phosphatidylinositol bisphosphate (PIP_2_) to two important second messengers, diacylglycerol (DAG) and inositol trisphosphate (IP_3_). In a similar way, phospholipase D (PLD) is activated, giving rise to the hydrolysis of phosphatidylcholine (PC) to DAG and choline. Lipophilic DAG remains in the proximity of the plasma membrane, being responsible for the activation of PKC with the participation of phosphatidylserine (PS) and Ca^2+^ ions. PKC activation involves phosphorylation and autophosphorylation with subsequent translocation of PKC to the plasma membrane in a process, involving the participation of annexins (anxs). In parallel, IP_3_ activates the IP_3_Rs on the surface of the sarcoplasmic reticulum (SR) allowing Ca^2+^ efflux. IP_3_ and DAG also activate the transient receptor potential canonical (TRPC) channels on the plasma membrane. The [Ca^2+^]_c_ elevation leads to the formation of the calcium-calmodulin (Ca^2+^-CaM) complex. The myosin light chain kinase (MLCK) becomes activated by the Ca^2+^-CaM complex resulting in subsequent phosphorylation of the myosin light chain (MLC) and initiation of contraction. The Ca^2+^-CaM complex also activates phosphatidylinositol-4,5-bisphosphate 3-kinase (PI_3_K), an enzyme that phosphorylates PIP_2_ to form another PKC activator, phosphatidylinositol trisphosphate (PIP_3_). Other pathways leading to PKC activation include oxidation, acetylation, or nitration. Once activated, PKC: 1) inhibits the K^+^ channels on the plasma membrane leading to depolarization, 2) directly activates the Ca_v_1.2 (L-type) calcium channels, 3) inhibits Na^+^/K^+^ ATPase, and 4) activates the Na^+^/H^+^ exchanger (NHE). Depolarization, Ca^2+^ influx and alkalinization of the cytosol promote contraction. In addition, PKC inhibits MLCP directly through the phosphorylation of MLCP, and indirectly through the phosphorylation of inhibitory proteins (CPI-17, PHI-1). PKC also phosphorylates proteins that bind actin in the unphosphorylated form (calponin, caldesmon), thereby allowing actin to bind myosin. PKC also inhibits soluble guanylate cyclase (sGC) and thus blocks the NO-induced vasodilatation. The effects of PKA are supported by parallel pro-vasoconstriction events: the Rho-associated protein kinase (ROCK) is activated by PI_3_K. DAG is also converted into arachidonic acid (AA) by the DAG lipase, which inactivates the MLCP directly and via the activation of ROCK. AA can also be produced after the activation of phospholipase A_2_ by the hydrolysis of phosphatidylethanolamine (PE). Other abbreviations: ADP, adenosine diphosphate; ATP, adenosine triphosphate; BK_Ca_, large conductance calcium-activated K^+^ channels; CPI-17, the phosphopeptide C-kinase potentiated protein phosphatase-1 inhibitor; GTP, guanosine triphosphate; IP_2_, inositol 1,4-bisphosphate; IP_3_R, inositol trisphosphate receptors; K_ATP_, ATP-dependent K^+^ channels; K_v_, the voltage-gated K^+^ channels; MLC-P, myosin light chain phosphorylated; NCX, sodium/calcium exchanger; P, phosphate; ↑pH, increase in cytosolic pH; PHI-1, phosphatase holoenzyme inhibitor; PKA, protein kinase A; PKC, kinase C; PKG, protein kinase G; PMCA, plasma membrane calcium ATPase; RyR, ryanodine receptor; SERCA, sarco/endoplasmic reticulum calcium ATPase

**Fig. 3:**
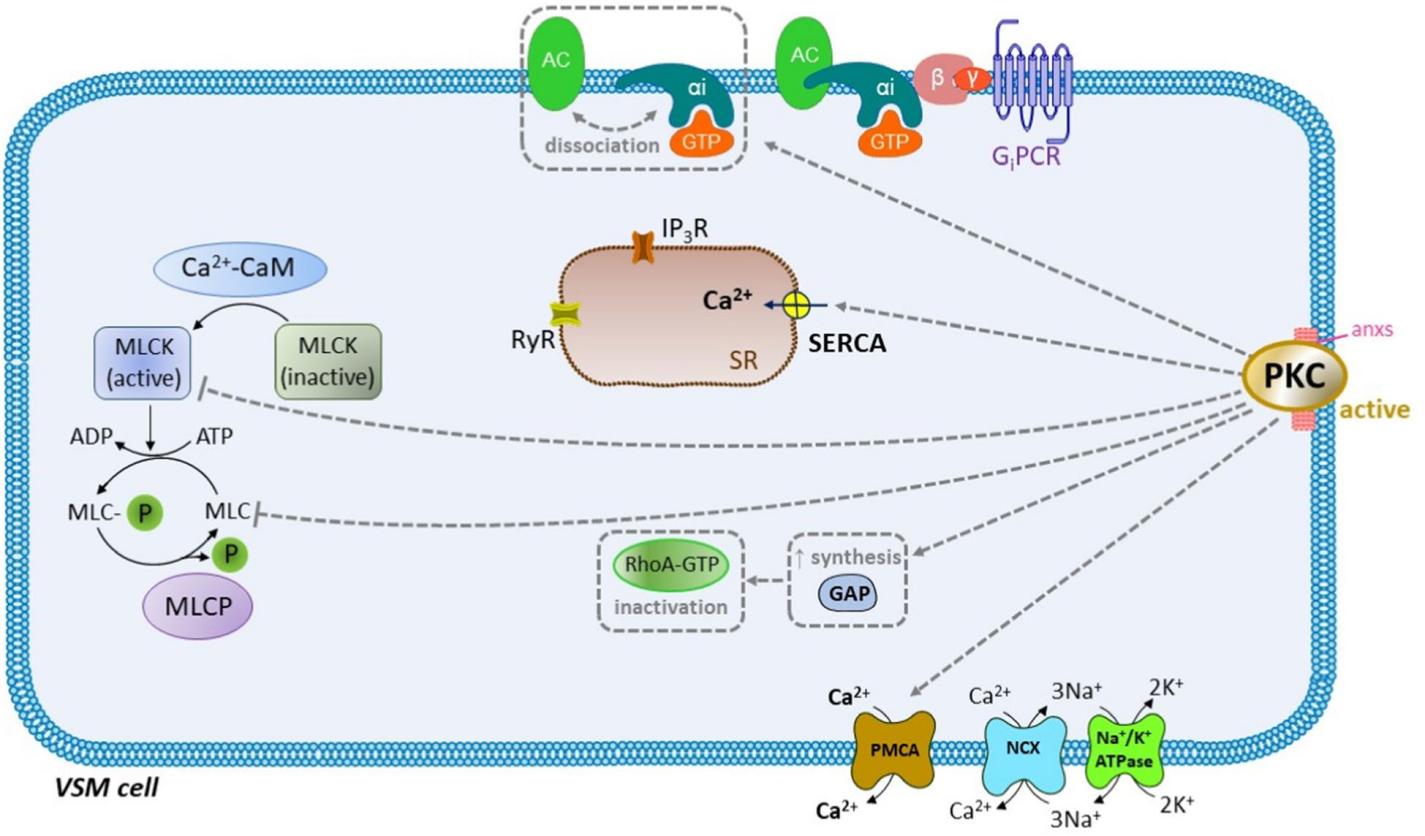
The dual role of protein kinase C (PKC) in the vascular smooth muscle (VSM) cells. Despite giving rise to vasoconstriction, PKC also acts in the opposite way securing a transient contraction. Among the counteracting effects of PKC are: 1) the inactivation of the myosin light chain kinase (MLCK); 2) the activation of the plasma membrane calcium ATPase (PMCA) and sarco/endoplasmic reticulum calcium ATPase (SERCA), giving rise to a drop in [Ca^2+^]_c_ and 3) enabling of the dissociation of the G protein-coupled receptor of the G_i_ type (G_i_PCR) from adenylate cyclase (AC), thereby terminating its inhibition. PKC also possibly enhances the synthesis of GTPase-activating protein (GAP), which is involved in the inactivation of RhoA-GTP. Other abbreviations: ADP, adenosine diphosphate; anxs, annexins; ATP, adenosine triphosphate; Ca^2+^-CaM, calcium-calmodulin complex; GTP, guanosine triphosphate; IP_3_R, inositol trisphosphate receptors; MLC, myosin light chain; MLC-P, myosin light chain phosphorylated; MLCP, myosin light chain phosphatase; NCX, sodium/calcium exchanger; NHE, sodium/proton exchanger; RyR, ryanodine receptors; SR, sarcoplasmic reticulum

The role of PKC is summarized in Figs. 2 and 3.

### RhoA/ROCK system

RhoA (Ras homolog family member A) is a small cytoplasmic GTPase from the Ras superfamily. Evolutionarily, RhoA is one of the oldest Rho GTPases. The protein has multiple functions, including the regulation of transcription and cell division. In the VSM cells, RhoA is abundant and significantly involved in vasoconstriction. The principle of functioning of this GTPase is the same as that of other small GTPases. In its inactive state, it is found in the cytoplasm in the form of RhoA-GDP bound to guanine nucleotide dissociation inhibitor (GDI).

#### Activation of RhoA and ROCK

RhoA is activated through two main pathways. First, by an extracellular vasoconstrictor, where [Ca^2+^]_c_ is not necessarily increased. Second, by elevated [Ca^2+^]_c_ with activation of PI_3_K by the Ca^2+^-CaM complex [198]. In the former case, an extracellular agonist binds GPCR of the G_12/13_-type and causes the dissociation of the α subunit. The RhoA-GDP dissociates from GDI and moves to the plasma membrane [94, 271]. The RhoA-GDP is activated upon activation of the guanine nucleotide-exchange factor (GEF), which exchanges GDP for GTP to give the active RhoA-GTP. The amount of RhoA-GTP is regulated by GTPase-accelerating protein (GAP), which stimulates the GTPase activity of G-protein (the α subunit catalyzes its own inactivation). Another mode of regulation is the phosphorylation of GDI by vasodilatory protein kinases A and G, which stabilizes the inactive Rho-GDP-GDI complex [27, 164, 217]. Importantly, the RhoA-GTP binds to the cytoplasmic Rho-associated protein kinase (ROCK), which becomes activated. In accord with the initial RhoA activation, the ROCK activation can occur with or without significant increase in [Ca^2+^]_c_ [83]. RhoA-independent ways of ROCK activation, such as that initiated by arachidonic acid, also exist [10].

#### ROCK and its effects

ROCK is a widely distributed cytoplasmic serine/threonine kinase. It has two isoforms, ROCK_1_ and ROCK_2_. After activation, ROCK moves partially to the plasma membrane. In general, the kinase exhibits vasoconstrictive effects as a result of the phosphorylation of many substrates. In particular, it phosphorylates the MYPT1 subunit (Thr695 and Thr853); the phosphorylation inactivates the MLCP and thereby prevents dephosphorylation of MLC_20_ [94, 179]. This is the most important effect of ROCK in the VSM cells, where the ROCK_2_ plays a major role [288]. The same process occurs indirectly through the CPI-17 (Thr38) [134] and through activation of the zipper interacting protein kinase (ZIPK), which subsequently phosphorylates the MYPT1 subunit (Thr696 and Thr18/Ser19). Interestingly, the CPI-17 is activated by PKC as well (see Sect."Effects of activated PKC"). Additionally, ROCK directly phosphorylates MLC_20_, which initiates muscle contraction [94, 179], and directly inactivates the K_v_ channels on the plasma membrane thus preventing its repolarization [169]. ROCK displays numerous other effects such as the phosphorylation of calponin [125], LIM kinases 1 and 2 [202, 258], ERM proteins (ezrin-radixin-moesin) [178] and mDia proteins (1 a 2) [49].

The RhoA/ROCK system influences the vessels also outside the VSM cells. Its inhibition increases NO release from the endothelial cells [148]. Inhibition of RhoA in the renal tubular epithelium may reduce the reabsorption of Na^+^ ions, which indirectly modifies the blood pressure [163]. In hypertension, the RhoA/ROCK system is implicated in the increase of the vascular stiffness via increased expression of vasoconstrictor proteins and increased peripheral resistance [107, 317]. Accordingly, a higher impact of the RhoA/ROCK has been reported in SHR in contrast to normotensive rats [115, 116]. The RhoA/ROCK activity is increased under vasospastic angina and cerebral vasospasm following subarachnoid hemorrhage, and the ROCK inhibitors such as fasudil have been shown to alleviate these spasms [146, 173].

#### The RhoA/ROCK feedback

Active ROCK inhibits its own activation by downregulating the GEF expression [33]. The ROCK inhibition decreases the secretion of acetylcholine [97] and increases the secretion of dopamine [303]. However, the latter effects have been reported outside the vascular system, and evidence for VSM is lacking.

**Fig. 4:**
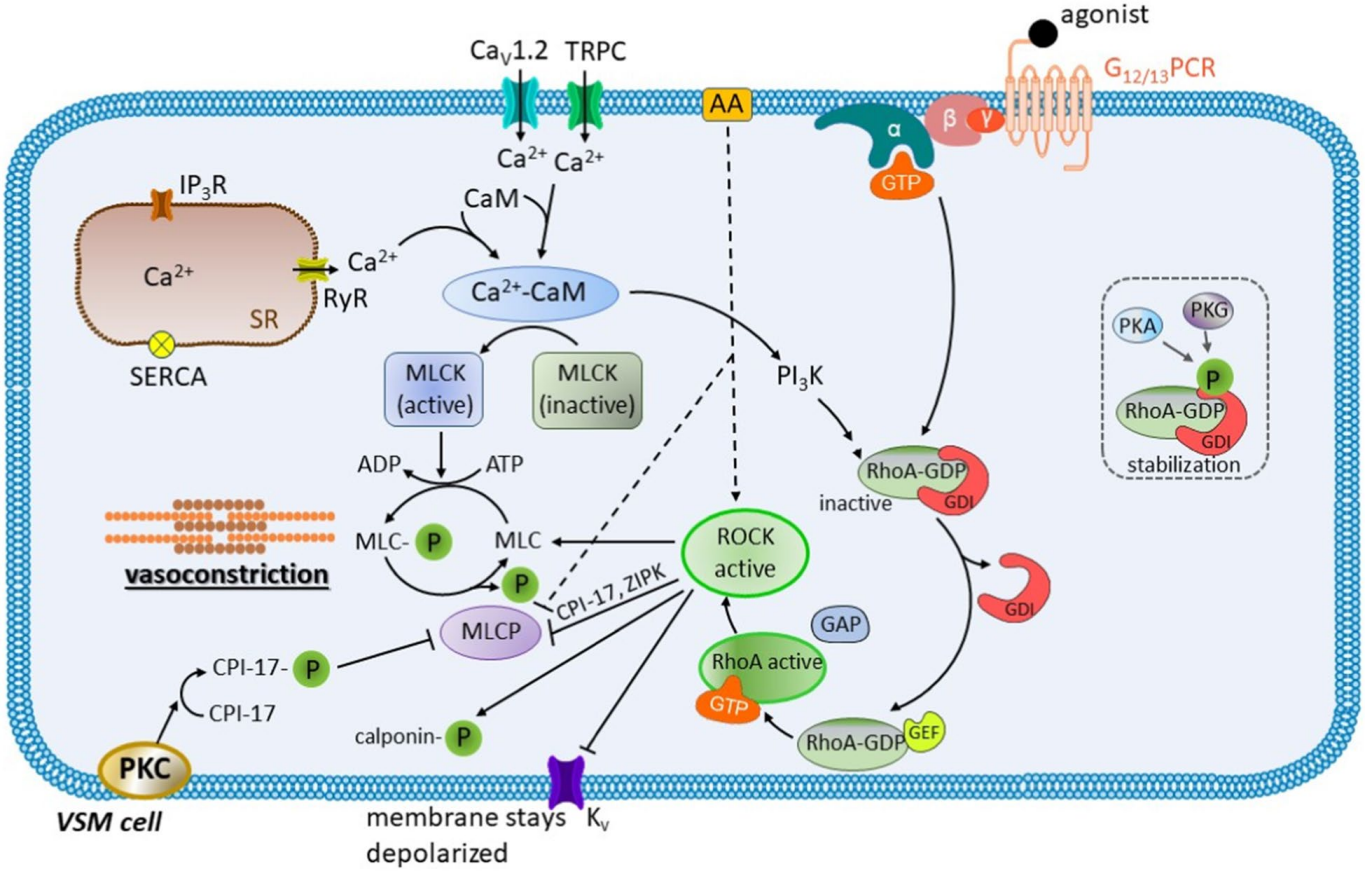
The role of RhoA/ROCK system in the vascular smooth muscle (VSM) cell contraction**.** In its inactive state, RhoA-GDP is bound to the guanine nucleotide dissociation inhibitor (GDI) in the cytoplasm. There are two main pathways which lead to its activation: 1) by an extracellular vasoconstrictor without triggering an increase in [Ca^2+^]_c_ and 2) by elevated [Ca^2+^]_c_ with the activation of phosphoinositide 3-kinase (PI_3_K) by the Ca^2+^-calmodulin complex (Ca^2+^-CaM). When an agonist binds to the G protein-coupled receptor of the G_12/13_ type (G_12/13_PCR) on the plasma membrane, dissociation of the α subunit takes place. Thereafter, RhoA-GDP dissociates from GDI and translocates to the plasma membrane, where the guanine nucleotide-exchange factor (GEF) exchanges GDP for GTP to form the active RhoA-GTP. The activation of RhoA-GDP can also be mediated through an increased [Ca^2+^]_c_ and subsequent formation of the Ca^2+^-CaM complex that activates PI_3_K. The GTPase-activating protein (GAP) stimulates the GTPase activity of the G-protein, and hence plays a crucial role in the regulation of the amount of RhoA-GTP. In addition, both protein kinase A (PKA) and G (PKG) are capable of phosphorylating GDI and consequently stabilize the inactive Rho-GDP-GDI complex. The active RhoA-GTP binds to the cytoplasmic Rho-associated protein kinase (ROCK) causing its activation. It is worthy to note that the activation of ROCK can occur with or without a significant increase in [Ca^2+^]_c_ and that arachidonic acid (AA) can also activate ROCK. Once active, ROCK phosphorylates various substrates thus giving rise to mainly vasoconstrictive effects. The ROCK effects include 1) inactivation of the myosin light chain phosphatase (MLCP) and consequent prevention of the dephosphorylation of the myosin light chain (MLC); 2) direct phosphorylation of MLC resulting in the initiation of muscle contraction, and 3) inactivation of the voltage-gated K^+^ (K_V_) channels on the plasma membrane, thus preventing K^+^ efflux (the membrane potential becomes more positive). ROCK also phosphorylates calponin, allowing actin to bind myosin in this way. In addition, protein kinase C (PKC) can also promote the inactivation of MLCP through the phosphorylation of the phosphopeptide C-kinase potentiated protein phosphatase-1 inhibitor (CPI-17) which also inhibits MLCP. Other abbreviations: ADP, adenosine diphosphate; ATP, adenosine triphosphate; Ca_v_1.2, L-type calcium channels; GDP, guanosine diphosphate; GTP, guanosine triphosphate; IP_3_R, inositol trisphosphate receptor; MLCK, myosin light chain kinase; MLC-P, myosin light chain phosphorylated; Na^+^/K^+^-ATPase, sodium/potassium ATPase; NHE, sodium/proton exchanger; NCX, sodium/calcium exchanger; RyR, ryanodine receptor; SERCA, sarco/endoplasmic reticulum ATPase; SR, sarcoplasmic reticulum; TRPC, transient receptor potential canonical channel; ZIPK, zipper interacting protein kinase

The role of RhoA/ROCK is summarized in Fig. 4.

## Endothelium in vasoconstriction

The endothelial cell monolayer, which separates VSM from the blood is localized in close proximity to the VSM. The monolayer is exposed to many chemical and physical stimuli. A sufficiently intense stimulus triggers the formation and release of endothelial vasoactive substances and/or changes in the membrane potential. Both have impact on the neighboring VSM and endothelial cells, and also act in an autocrine fashion. Intercellular gap junctions formed by connexins play an important role in transmitting the changes in the membrane potential and possibly some of the small signaling molecules such as Ca^2+^, IP_3_ and small molecules <  ~ 1 kDa [114, 188]. As regards the former, the movement of the calcium ions through the gap junctions can be slower than other calcium signaling pathways, and it is unclear to what extent this accounts for the intercellular communication. The gap junctions between the smooth muscle and endothelium (MEGJ) contribute to myoendothelial communication. Similarly, the gap junctions between the adjacent endothelial cells (EEGJ) mediate communication in the axial direction. The importance of the gap junctions increases with decreasing arterial diameter. Conversely, their number and cell-to-cell communication decrease with age, and the expression of particular connexins is altered during senescence [308]. Other factors such as hyperlipidemia [155] or smoking [218] can also affect endothelial connexin expression.

Depolarization of the endothelial cell membrane promotes vasoconstriction, whereas hyperpolarization promotes vasodilatation [129, 281, 290]. Vasoactive substances from the endothelium are divided into the endothelium-derived contracting factors (EDCFs), and the endothelium-derived relaxing factors (EDRFs). Importantly, the role of the endothelial Ca^2+^ ions is different from their role in the VSM cells. While a global increase of Ca^2+^ concentration in the VSM cells leads to vasoconstriction, inside the endothelial cells, calcium induces vasodilatation due to the production of EDRFs and induction of hyperpolarization. As the aim of this review is to summarize the machinery of vasoconstriction, the principal EDCFs will be described next.

### Endothelium-derived contracting factors EDCFs

Endothelium produces EDCFs which influence the adjacent VSM and endothelial cells. The best known EDCF is endothelin-1, although vasoconstrictive prostanoids might be more important. The following sections provide information on endothelin-1, vasoconstrictive prostanoids, hydroxyeicosatetraenoic acids, and some other EDCFs.

#### Endothelin 1

Endothelin 1 (ET-1) is a small peptide (21 AK) synthesized from big-ET-1 by the endothelin converting enzymes (ECEs). The ET-1 acts through the GPCR receptors called ET_A_ and ET_B_. Both are located on the plasma membrane of VSM, where the ET_A_ subtype dominates; the activation of the proteins gives rise to vasoconstriction. The ET_B_ receptors are also present on the endothelial cells, where they are linked to vasodilatation. Upon being released from the endothelial cells, ET-1 induces a biphasic response. First, a short-term drop in the blood pressure caused by the activation of the endothelial ET_B_ receptors of G_q_ and G_i_ types with rise in both NO and PGI_2_ (prostacyclin) production occurs. The initial response is followed by vasoconstriction, mainly due to ET_A_ receptor activation on the VSM [267], mediated by at least two pathways: the receptor of the G_q_ type (PKC activation) and that of the G_12/13_ type (RhoA/ROCK activation). The ET_B_ receptors on the smooth muscle are also involved in vasoconstriction.

Due to rapid elimination, the effects of ET-1 are mainly local [266]. Under physiological conditions, the amount of ET-1 is tightly regulated. Upon reaction with the endothelial ET_B_ receptors, ET-1 itself increases the production of NO, which inhibits ECEs [159]. Calcitonin gene-related peptide (CGRP) is another regulator, which promotes the dissociation of ET-1 from the ET_A_ receptor. The CGRP is produced by the perivascular sensory neurons and may act through the membrane receptor and βγ subunits of the G-protein [181]. The CGRP can be released by capsaicin through the TRPV_1_ channel activation (reported in the rat mesenteric artery) [128]. In addition, the CGRP activates the K_v_ channels and plasma membrane hyperpolarization.

Under physiological state, pharmacological blockade of the endothelin receptors has a mild effect. The importance of ET-1 increases under hypoxia [220] and pathological conditions. Its constitutive production is increased in diabetic patients, who have elevated ET-1 plasma levels and develop hyperresponsiveness to its effects, which contributes to endothelial dysfunction [174]. In hypertensive patients, ET-1 increases the tone of the afferent and efferent glomerular arterioles, decreases glomerular filtration rate [142], exhibits proinflammatory and profibrotic effects, promotes vascular remodeling and increases ROS production [238]. However, ET-1 plasma level is not necessarily elevated in the hypertensive patients, perhaps due to efficient elimination [79]. The use of ET-1 antagonists in routine clinical practice has so far been limited by their adverse effects, such as peripheral edema and reproductive toxicity in animals. They have been applied to the treatment of pulmonary hypertension (ambrisentan, bosentan, macitentan) [54, 208] and hypertension (aprocitentan) [56]. Interestingly, ET-1 is also involved in the development of atherosclerosis, myocardial infarction and heart failure [119, 138]. Its role may be dual [138], and possible pharmacological intervention is not yet resolved.

#### Prostanoids causing vasoconstriction

For vasoconstriction, the endothelium-originated prostanoids that diffuse to the VSM cells are more important than those produced directly in the VSM cells. COX-1 is their main producer under physiological conditions, whereas the importance of up-regulated COX-2 increases under hypertension, diabetes and obesity [284, 305]. COX-2 expression also increases with age; contribution of a more intense shear friction during pulsatile blood flow is also possible [269]. The role of prostanoids varies depending on the species and vascular beds. Vasoconstrictive effects have so far been demonstrated for PGH_2_ and its derivatives PGF_2α_ and thromboxane A_2_ (TxA_2_) [78, 268, 294]. The production of TxA_2_ was classically associated only with platelets, but, under some conditions, it may also be produced in the endothelium, as described in the aorta of SHR [78]. On the VSM cells, these three prostanoids activate the GPCR of the G_q_-type: FP (PGF_2α_) and TP (TxA_2_, PGH_2_) [192, 305]. The resultant effects are determined by the interplay among the mediators present, especially between the vasoconstrictive TxA_2_ and the vasodilatory PGI_2_. Surprisingly, mediators such as PGI_2_ or NO can give rise to vasoconstriction under some conditions [71, 78, 109] (see Sect."Endothelial vasoconstriction counteracting previous stimuli"). Last, isoprostanes (IsoPs) are prostaglandin-like compounds the levels of which are elevated in a number of diseases [227]. The IsoPs are produced by the free radical-catalyzed peroxidation of arachidonic acid independent of the COX [194]. The 15-F_2t_-IsoP (also known as 8-isoPGF_2alfa_) is a potent vasoconstrictor acting via the TP receptor in several vascular beds [105, 193]. The same substance also increases endothelin 1 release, induces VSM cell proliferation and may inhibit platelet aggregation [207].

#### Hydroxyeicosatetraenoic acids

The vasoconstriction caused by the hydroxyeicosatetraenoic acids such as the 20-HETE is worth mentioning. This eicosanoid is the metabolite of arachidonic acid produced by the CYP4A and CYP4F enzymes. It is another CYP metabolite of arachidonic acid in addition to the epoxyeicosatrienoic acids (EETs), but with opposite effect on the vessels. 20-HETE is a TP receptor agonist [70]. Its vasoconstrictive action is mediated by activation of PKC and ROCK [203, 221, 229]. On the plasma membrane, the Ca_v_1.2 [309, 312] and TRPC_6_ channels [113, 232] are activated, and the BK_Ca_ channels inhibited [319]. Even though 20-HETE is generally viewed as a vasoconstrictor, opposite effects were also reported in some vascular beds including rabbit kidney [34], bovine coronary [215], bovine pulmonary [310] and mice basilar [62] arteries. These effects were partially blocked by indomethacin, indicating an involvement of cyclooxygenase, and by the removal of endothelium. The vasodilatation was attributed to NO synthetized by the endothelial NO synthase (eNOS) and to PGI_2_. The formation of the latter might have resulted from the increased release of arachidonic acid due to the elevation of [Ca^2+^]_c_ triggered by 20-HETE [62]. Despite this contradiction, 20-HETE is generally regarded as a factor contributing to vascular dysfunction, and the development of hypertension [104]. Noteworthy, 20-HETE is interlinked with the renin/angiotensin system. Angiotensin II induces 20-HETE synthesis and release. Reciprocally, 20-HETE increases angiotensin-converting enzyme (ACE) transcription in the endothelial cells [104]. The substance also eliminates the endothelial effects of insulin [157].

#### Other pro-vasoconstrictive factors

Obesity, insulin resistance and diabetes [174], hypertension and age are the key factors that promote endothelium-dependent (EDCF) vasoconstriction [18, 205, 280, 282]. This conclusion is supported by several studies. In obese sedentary mice, the microvascular dysfunction, inflammation and ROS production were observed [80]. In obese subjects and diabetics, the production of endothelial ET-1 was elevated [174]. In spontaneously hypertensive rats, the calcium handling dysfunction (higher expression of the IP_3_R_2_ channels and SERCA_3_) and impaired endothelial calcium signaling were reported in the aortic endothelial cells [195, 291]. Change in endothelial [Ca^2+^]_c_ levels under hypertension is thus possible, but there is not a clear consensus. Both higher [262] and lower [287] calcium levels were found in the aortic endothelial cells from spontaneously hypertensive rats compared to normotensive controls. However, this discrepancy could be related to the methodological difference (fresh endothelial cells vs cultured cells). Ageing is linked to COX up-regulation and increased ROS levels [205], and to affected calcium signaling [92]. Vasoconstriction by EDCFs may be intensified under vitamin D deficiency [292, 293] which is more common in seniors. Endothelial dysfunction is also promoted by many indirect factors, such as increased arginase activity [246] or deficiency of tetrahydrobiopterin (BH_4_, required for the production of NO) [3].

While the detailed course of pronounced vasoconstriction under obesity, diabetes, hypertension and ageing can differ from case to case, ROS production is increased under all these conditions. The COX is the main source of the endothelial ROS [262] (there are also other sources, including the monomeric eNOS, and the ROS are also generated in PVAT [286] [80]). The ROS are vasoactive in several ways. First, eNOS dimerization and NO formation is inhibited [67, 158]. Second, superoxide anions (O_2_^.−^) react with the NO present to form peroxynitrite (ONOO^−^) which is vasoactive. Its effects are concentration-dependent. While peroxynitrite at very low concentrations (1–10 nM) activates COX and elevates H_2_O_2_ levels, the prostacyclin synthase is inactivated by nitration and PGI_2_ production decreased at 10–20 folder higher levels. Consequently, the PGE_2_ and PGF_2α_ production predominates [77]. Its effects on platelet aggregation are also dual, and switch from anti-aggregatory to pro-aggregatory with higher peroxynitrite levels [247]. Third, under redox stress, when the cellular glutathione disulfide/glutathione ratio is high, the eNOS becomes uncoupled by glutathionylation, and produces superoxide [96]. Fourth, the ROS diffuse into the VSM cells, where they activate the COX and the production of vasoconstrictive prostanoids (see Sect."Prostanoids causing vasoconstriction") [244]. And, finally, an excessive ROS production dysregulates the Ca^2+^ signaling in general [199].

### Endothelial vasoconstriction counteracting previous stimuli

Although vasodilatation physiologically predominates over vasoconstriction at normal resting tone, the counteracting mechanisms are triggered in parallel. The endothelium-derived contraction can be also triggered by a sudden or severe stretching of a vessel [127] (see Sect."Mechanical stimuli"). These negative feedbacks are very important for the integrated vascular function, and endothelium plays a crucial role. By way of example, the agonists of the endothelial GPCR of the G_q_ type, such as acetylcholine (receptor subtype depends on the vascular bed, e.g. the M_3_ receptors predominate in coronary circulation, while the M_5_ receptors predominate in CNS [144, 301]) or ATP (P_2_Y receptors) primarily induce endothelium-dependent hyperpolarization (EDH) and vasodilatation. They also display counteracting effects, including the activation of the Ca^2+^ independent iPLA_2_. The produced lysophospholipids open the store-operated Ca^2+^ channels, and the subsequent Ca^2+^ influx activates cPLA_2_. Arachidonic acid is released and COX-derived EDCFs generated [281]. The formation of individual prostanoids depends on the availability of prostaglandin synthases (TxA_2_ synthase for TxA_2_) and their activities, which are significantly modulated by ROS (see Sect."Other pro-vasoconstrictive factors").

In general, the RhoA/ROCK pathway counteracts the NO vasodilatation [216]. This cascade decreases the activity of PKB which is the direct activator of eNOS by phosphorylation (Ser-1177) [307]. It enhances arginase activity and thereby limits the amount of *L*-arginine for NO synthesis [187]. Furthermore, increased activity of the arginases gives rise to eNOS-uncoupling and thus switches its activity from the production of NO to that of the superoxide [306]. Accordingly, increased activity of the endothelial RhoA/ROCK pathway is associated with endothelial dysfunction [245]. However, its physiological function is undisputable as the pathway participates in the mechanosensing [180] of the natural blood flow and of the vascular stretch resulting in pressure-induced (myogenic) response (see Sect."Mechanical stimuli"); and is necessary for the endothelial barrier integrity [219]. Notably, low concentrations of angiotensin II stimulate calcium sparks [11].

Under some (non-physiological) conditions, even some vasodilators exhibit vasoconstrictive effects. Under hypoxia, NO induces coronary artery contraction, which is sGC-dependent, but unrelated to cGMP. Cyclic inosine monophosphate (cIMP) is produced, and this effect is associated with the activation of ROCK [71, 109]. Analogously, PGI_2_ may induce vasoconstriction, and its elevated levels were found in SHR together with elevated TxA_2_ [78]. The IP receptors that physiologically mediate the PGI_2_ vasodilatory effects are unlikely to be functional. In contrast, the TP receptors show elevated sensitivity to PGI_2_ which results in vasoconstriction instead of vasodilatation [64]. Similarly, acidification of the endothelial and VSM cells surprisingly inhibits eNOS activity [25].

**Fig. 5:**
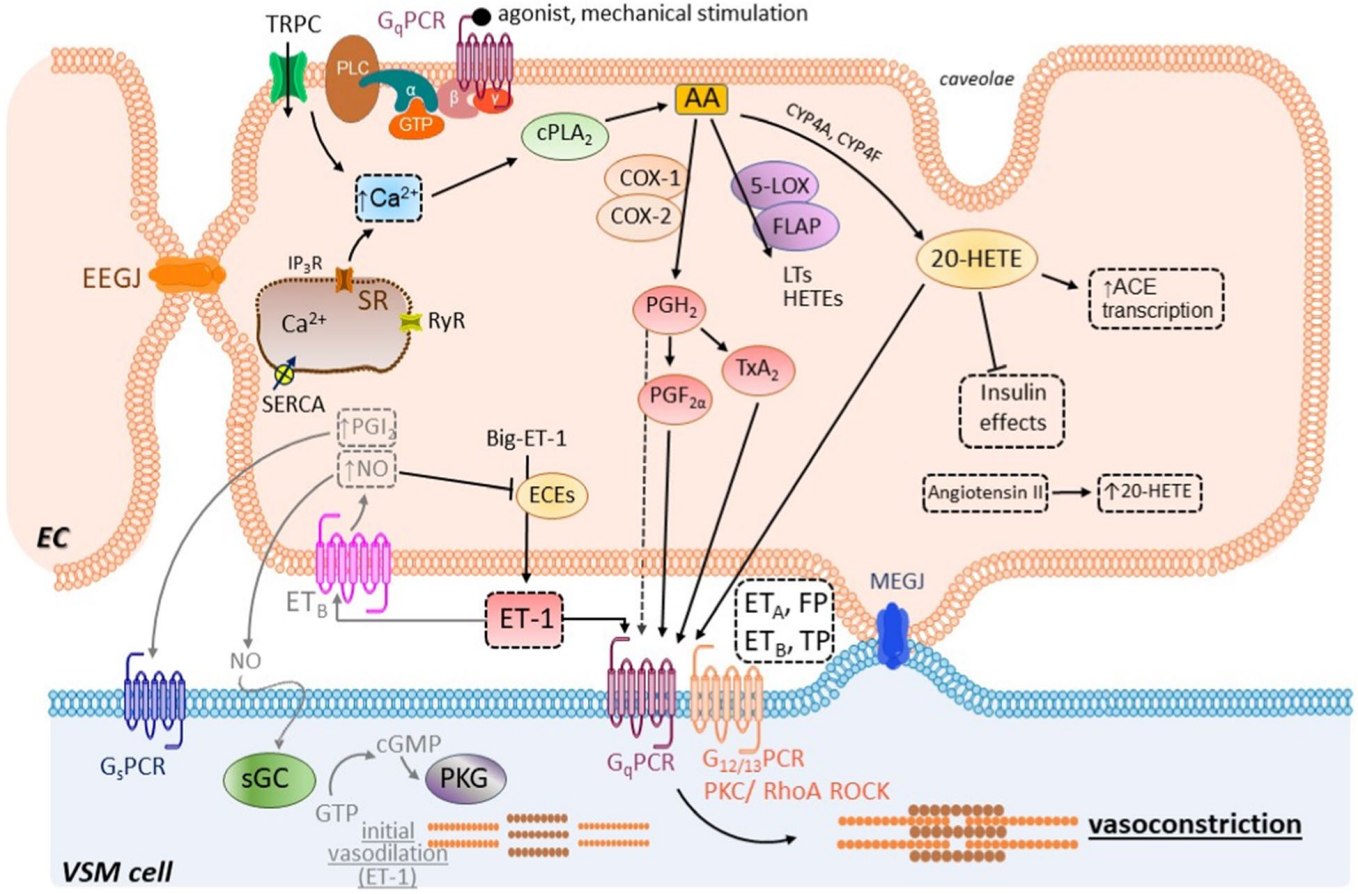
Schematic representation of the endothelium-dependent vasoconstriction. The following are among the endothelium-derived constrictor factors (EDCFs): endothelin-1 (ET-1), vasoconstrictor prostanoids, and hydroeicosatetraenoic acids (HETEs). ET-1 is a small peptide produced from big-ET-1 by the endothelin converting enzymes (ECEs). There are two types of ET-1 receptors, namely the ET_A_ and ET_B_ receptors. Both ET_A_ and ET_B_ receptors are located on the plasma membrane of the VSM, where the ET_A_ receptors predominate. Both types mediate vasoconstriction. The ET_B_ receptors are also present on the plasma membrane of the endothelial cells, but, in this case, they give rise to vasodilatation. After being released from the endothelial cells, ET-1 induces a biphasic response that is composed of an initial vasodilatory effect, followed by vasoconstriction. First, ET-1 activates the endothelial ET_B_ receptors with an increase of nitric oxide (NO) and prostacyclin (PGI_2_) production. Both NO and PGI_2_ diffuse to the VSM and exhibit their vasodilatory effects via the soluble guanylate cyclase/protein kinase G (sGC/PKG) and the IP(G_s_)/AC/cAMP receptor. Following this initial response, vasoconstriction takes place, mainly due to the activation of the ET_A_ receptors on the vascular smooth muscle (VSM) cell through at least two pathways: the G protein-coupled receptor of the G_q_ type/phospholipase C/inositol trisphosphate + diacylglycerol (G_q_/PLC/IP_3_ + DAG) and G protein-coupled receptor of the G_12/13_ type/RhoA/Rho-associated protein kinase (G_12/13_/RhoA/ROCK) pathways. The ET_B_ receptors on the VSM are also partially involved in vasoconstriction. The vasoconstrictor prostanoids and 20-HETE activate the FP and TP receptors on the VSM cell. Other abbreviations: AA, arachidonic acid; ACE, angiotensin converting enzyme; BK_Ca_, large conductance calcium-activated K.^+^ channel; Ca_v_1.2, L-type calcium channels; cGMP, cyclic guanosine monophosphate; cPLA_2_, cytosolic phospholipase A_2_; COX, cyclooxygenase; EC, endothelial cell; EEGJ, gap junctions between adjacent endothelial cells; FLAP, 5-lipoxygenase activating protein; FP, PGF_2α_ receptor; GTP, guanosine triphosphate; IP_3_R, inositol triphosphate receptors; 5-LOX, 5-lipoxygenase; LT, leukotrienes; MEGJ, myoendothelial gap junctions; PKC, protein kinase C; PLC, phospholipase C; RyRs, ryanodine receptors; SERCA, sarco/endoplasmic reticulum calcium ATPase; SR, sarcoplasmic reticulum; TP, thromboxane A_2_ receptor; _TRP_C, transient receptor potential canonical channel; TxA_2_, thromboxane A_2_

The role of the endothelium in vasoconstriction is summarized in Fig. 5.

## Vasoconstrictive stimuli

The endothelium and the VSM cells sense vasoactive stimuli. The interplay between them modifies the membrane potential and the traffic of Ca^2+^ ions, which are decisive for the smooth muscle response. The following sections deals with principal vasoconstrictive stimuli and their effects. According to origin, these stimuli can be divided into physical and chemical. The physical stimuli can be categorized as mechanical and thermal.

### Mechanical stimuli

The mechanical stimuli are always in operation, even under the resting conditions as a result of the blood flow and physiological intravascular pressure. Physiologically, vasodilatation prevails over vasoconstriction due to the fluid shear stress and pulsatile stretch of the vascular wall with stimulation of NO production. In small arteries, elevated intraluminal pressure results in myogenic response, which contributes to the basal vascular tone and is important for the autoregulation of the blood flow. In mouse small mesenteric and renal arteries ex vivo*,* this response was observed after the elevation of the intraluminal pressure above 60 mmHg [248]. The myogenic response starts with the detection of the vascular wall stress. The mechanosensing is likely secured in multiple ways, and there are several candidates the mechanosensors, such as some GPCR and TRP channels. The AT_1_ receptors were reported to be mechanosensitive, first in the cardiomyocytes [320] and leter in other vascular beds, such as murine mesenteric or renal arteries [248]. The essential involvement of the AT_1a_ receptor subtype was found in the same study. The activation of the AT_1_ receptors triggers the G_q_/PLC/IP_3_ + DAG/PKC cascade (see Sect."Protein kinase C") [46]. Various TRP channels (formerly referred to as the stretch-activated channels, SACs) are involved in the myogenic response. However, there is little evidence supporting their direct activation by stretching in most cases, and the TRP channels can be rather viewed as the downstream members of the myogenic response. For example, the TRPC_6_ channels are activated downstream of the mechanosensitive GPCR including the AT_1_ [180]. Similar results were found for the TRPM_4_ [211] and TRPV_1_ [210] channels. On the other hand, the TRPP_1_ [241] and TRPV_4_ channels [260] may be mechanosensitive when associated with other proteins, but more experiments are needed to unravel their role. The endothelial TRPC_6_ channels were also found to be mechanosensitive, but the results were obtained on cultured cells [315]. Last, the membrane glycoproteins-integrins [168] and the platelet endothelial cell adhesion molecule PECAM-1 [124] can also be involved. After mechanosensing, the plasma membrane is depolarized which leads to the opening of the Ca_v_1.2. channels and Ca^2+^ influx. Elevation of [Ca^2+^]_c_ triggers vasoconstriction [136]. The transient response is secured by negative feedback mechanisms with the return of the membrane potential and [Ca^2+^]_c_ to the resting values (see Sect."The Ca^2+^ feedback"). The adipose-derived relaxing factor from PVAT may also participate in the tone regulation [81, 263]. There are obvious differences among specific arterial beds, for example, the myogenic response is more pronounced in the cerebral arteries than in the mesenteric arteries [143]. Other factors, such as species, age [45], and general health state have also an impact. An impairment in the myogenic response can be related to the progression of many diseases including diabetes [137, 250], heart failure [152] and stroke [40]. While the myogenic tone caused by fluid shear stress is an important part of vascular physiology, the same is not true for strong vessel stretching resulting from high arterial pressure. High arterial pressure can induce vascular smooth muscle hypertrophy and hyperplasia with changes in the contractile and matrix proteins and is involved in vascular pathologies [8]. Notably, cardiac pulsation can generate circumferential stretch [32] which leads to angiotensin II release from the endothelium and the elevation of superoxide levels [167], the superoxide may thus be involved in the signaling pathways. The effects on health can be both positive and negative [8, 9, 102].

### Thermal stimuli

If the temperature drops under a certain level, the organism responds by vasoconstriction. Mild hypothermia may be detected mainly by endothelium, while the temperature below approx. 30 °C may directly affect the smooth muscle. The vascular response to temperatures below 20 °C was endothelium-independent [60]. Various TRP channels are thought to be temperature sensitive. The TRPV_3_ and TRPV_4_ channels are highly sensitive to temperatures around 30 °C. The TRPM_8_ channels opening at temperatures lower than 20 °C are the best understood cold-sensitive TRP channels. The mammalian TRPA_1_ may be another cold sensor which opens at temperatures below 10 °C [316]. Different vessels response differently to temperature changes. For example, the canine coronary artery responded to hypothermia endothelium-independently, while the canine renal and femoral arteries endothelium-dependently [60].

### Chemical stimuli

Chemical stimuli have either intracellular or plasma membrane targets. The latter are often associated with the GPCR. The resultant effect depends not only on the type of the G protein and the corresponding signaling pathway, but also on the location of the receptor (endothelium vs the smooth muscle). Importantly, the same receptor types can be located on the endothelium, the smooth muscle, or both sites. The activation of the endothelial G_12/13_ and the smooth muscle G_q_, G_i_ and G_12/13_ receptors gives rise to vasoconstriction. In contrast, the endothelial G_q_, G_s_ and G_i_ receptors and the G_s_ receptors of the smooth muscle mediate vasodilatation. This means that particularly for G_q_ and G_i_ receptors, it is necessary to distinguish whether they are located on the endothelium, the smooth muscle or both sites. After the activation of the GPCR, the intracellular pathways in the endothelium are analogous to those in the smooth muscle. Last but not least, one substance can act on more receptor types and one receptor type can be linked to more than one intracellular cascade. The complexity of the vasomotor action can be demonstrated using angiotensin II as an example. The vascular effects of angiotensin II are mainly mediated by the AT_1_ receptor. Its agonists activate both the G_q_/PLC/IP_3_ + DAG/PKC and the G_12/13_/RhoA/ROCK cascades. In the VSM cells, both cascades promote vasoconstriction. In the endothelium, the G_12/13_ pathway is also vasoconstrictive, e.g. the synthesis and release of 20-HETE are thus induced. In contrast, the activation of the endothelial G_q_ pathway with the activation of eNOS is vasodilatory. Accordingly, the endothelial AT receptors (possibly the AT_1a_ subtype) are susceptible to the blood-flow induced shear friction, and angiotensin II at low concentrations can trigger the calcium sparks which are important in protection of small resistance arteries against excessive pressure [11, 180]. Other vasoconstrictive substances influence the membrane ion channels directly (e.g. an activator of Ca_v_1.2 channels, Bay K8644, or BK_Ca_ channel blocker, iberiotoxin) or enter the cells and target the components of intracellular signaling pathways, such as cyclases (an inhibitor of soluble guanylate cyclase, ODQ), protein kinases (an activator of PKC, bryostatin 1), ion channels on the SR (an activator of RyR channels, caffeine), or SERCA (an inhibitor, thapsigargin).

## Conclusions

The vascular system controls the distribution of blood and the substances dissolved into all body tissues. Together with the heart, the system secures the circulation of blood, which is essential for life. Vasoconstriction and vasodilatation are two important mechanisms to rapidly and efficiently regulate blood flow. Vasoconstriction is sometimes perceived rather negatively because is associated with endothelial dysfunction [223]. This is not quite correct, as vasoconstriction represents an essential part of normal vascular physiology. The adequate vasoconstriction prevents excessive vasodilatation and secures the blood flow and pressure, necessary to supply blood to tissues. This function is guaranteed by several vasoconstrictor signaling pathways that are interconnected in multiple ways. At the same time, they are interconnected with vasodilator mechanisms tightly regulating each other. The close interdependence of the vascular smooth muscle with the vascular endothelium plays prominent role. Perfect orchestration of all components is necessary for vascular homeostasis, guaranteeing quick and efficient responses to immediate need under various circumstances.

## Data Availability

No datasets were generated or analysed during the current study.
